# Community-Wide Spatial and Temporal Discordances of Seed-Seedling Shadows in a Tropical Rainforest

**DOI:** 10.1371/journal.pone.0123346

**Published:** 2015-04-09

**Authors:** Débora Cristina Rother, Marco Aurélio Pizo, Tadeu Siqueira, Ricardo Ribeiro Rodrigues, Pedro Jordano

**Affiliations:** 1 Departamento de Botânica, Universidade Estadual Paulista, Rio Claro, São Paulo, Brazil; 2 Departamento de Zoologia, Universidade Estadual Paulista, Rio Claro, São Paulo, Brazil; 3 Departamento de Ecologia, Universidade Estadual Paulista, Rio Claro, São Paulo, Brazil; 4 Departamento de Ciências Biológicas, Laboratório de Ecologia e Restauração Florestal, Escola Superior de Agricultura Luiz de Queiroz, Universidade de São Paulo, Piracicaba, São Paulo, Brazil; 5 Integrative Ecology Group, Estación Biológica de Doñana, Consejo Superior de Investigaciones Científicas, Isla de La Cartuja, Sevilla, Spain; University of New South Wales, AUSTRALIA

## Abstract

Several factors decrease plant survival throughout their lifecycles. Among them, seed dispersal limitation may play a major role by resulting in highly aggregated (contagious) seed and seedling distributions entailing increased mortality. The arrival of seeds, furthermore, may not match suitable environments for seed survival and, consequently, for seedling establishment. In this study, we investigated spatio-temporal patterns of seed and seedling distribution in contrasting microhabitats (bamboo and non-bamboo stands) from the Brazilian Atlantic Forest. Spatial distribution patterns, spatial concordance between seed rain and seedling recruitment between subsequent years in two fruiting seasons (2004–2005 and 2007–2009), and the relation between seeds and seedlings with environmental factors were examined within a spatially-explicit framework. Density and species richness of both seeds and seedlings were randomly distributed in non-bamboo stands, but showed significant clustering in bamboo stands. Seed and seedling distributions showed across-year inconsistency, suggesting a marked spatial decoupling of the seed and seedling stages. Generalized linear mixed effects models indicated that only seed density and seed species richness differed between stand types while accounting for variation in soil characteristics. Our analyses provide evidence of marked recruitment limitation as a result of the interplay between biotic and abiotic factors. Because bamboo stands promote heterogeneity in the forest, they are important components of the landscape. However, at high densities, bamboos may limit recruitment for the plant community by imposing marked discordances of seed arrival and early seedling recruitment.

## Introduction

The seed-seedling transition is the least predictable stage of plant recruitment [1], [2]. Such unpredictability occurs because early stages of the recruitment process are especially variable and prone to density-dependent effects at various spatial and temporal scales [1], [3]. On a local spatial scale, plant population dynamics are largely determined by seed arrival [4], a process influenced by spatially variable factors such as plant fecundity and seed dispersal [3] and how they interact with complex landscapes. The place where an adult plant is established affects its seed production [3], while the structure of surrounding environment can influence seed dispersal. For animal-dispersed species, for instance, the disperser behavior, habitat preferences and predator avoidance are biotic influences that can lead to highly aggregated seed deposition in specific environments, with lasting consequences for the subsequent stages of recruitment [5], [6], [7]. For wind-dispersed seeds in turn, forest gaps may function as seed traps [8]. After the seeds reach a given microsite, there are multiple abiotic (e.g., nutrient availability, water and light) and biotic factors (e.g., seed predators, herbivores and pathogens), which will further affect the post dispersal stages of recruitment.

Abiotic and biotic factors acting independently of each other and upon each stage of the recruitment process may lead to high context-dependency of the recruitment success [9]. This context-dependency largely results from variable qualities of specific microhabitats for different recruitment stages, leading to spatial discordances between the initial seed rain and final recruitment [10], [11]. When seed rain is more heterogeneous than seedling establishment, concordance occurs between the two recruitment stages, whereas discordance occurs when seedling establishment is more spatially heterogeneous than seed rain [12]. Accordingly, environments that combine high seed rain with high success of seedling establishment may be considered important *hotspots* of plant recruitment [13]. Although single-species studies have been conducted in species-rich tropical forests (e.g., [14]), a lasting challenge to understand tropical forest dynamics has been to unveil the multiple, delayed effects acting upon plant recruitment with a community-wide perspective.

In this paper we contrasted the spatial patterns of seed dispersal and recruitment sites in two fruiting periods and two different habitats represented by bamboo and non-bamboo stands in an area of Brazilian Atlantic rainforest where *Guadua tagoara* (Nees) Kunth, a native bamboo species, created a marked patchiness and heterogeneity in the vegetation [15]. We emphasize the importance of studying seed and seedling spatial patterns across different habitats and periods as essential for understanding the complete sequence of processes involved in plant recruitment stages in a tropical forest mosaic. Moreover, our approach combines soil and stand characteristics. Specifically, our key questions were: (*i*) do the spatial patterns of seed deposition and seedling establishment differ between bamboo and non-bamboo stands? Considering two sampling periods and bamboo and non-bamboo stands, (*ii*) is there spatial and temporal concordance in seed and seedling density and species richness? Moreover, (*iii*) are there specific abiotic environmental characteristics associated with *hotspots* of recruitment? and (*iv*) are *hotspots* of recruitment spatially related to either bamboo or non-bamboo stands?

## Materials and Methods

### Study site

The study site was located in the lower montane forest of Carlos Botelho State Park (PECB) (24°10’S, 47°56’W), a reserve that is part of one of the largest and well-preserved blocks of Atlantic Forest in Brazil with over 120,000 ha. The Technical Scientific Committee (COTEC) from Instituto Florestal (IF-SP) issued all of the required permits for the work conducted in PECB.

The study took place at a permanent plot located at 300 m asl on the Atlantic slope of the Serra de Paranapiacaba mountain range. The plot was a 320 x 320 m quadrat divided into a grid of 256 sub-plots of 400 m^2^ each, totaling 10.24 ha [16]. Soil, topography, climate, and light were abiotic characteristics surveyed within each sub-plot. Mean temperature during the study period was 21.1°C (range 17.4–25.2°C), and mean rainfall was 3,384 mm.

The vegetation was composed of high (20–30 m), old-growth lowland forest, with an opened understory interspersed by stands of the native bamboo *Guadua tagoara* [17], an endemic species of the Brazilian Atlantic forest [18]. *G*. *tagoara* have green, hollow and scandent culms, with heights and girths of 8–15 m and 5–10 cm, respectively [19]. Their numerous culms rely on the surrounding vegetation aided by their recurved thorns in the nodes [20]. The species is monocarpic with synchronized flowering [21]. After its fruiting event, the stand of this bamboo species dies. Here, we consider individual as a culm of a stand. Almost one third of the permanent plot is covered by *G*. *tagoara* which forms stands where the great density of culms makes hard to walk through. The overabundance of this bamboo species in the study region is related to a set of causes such as the absence of large herbivores and other human-mediated disturbances [20]. Apart from bamboos, the old-growth forest understory is dominated by the palm *Euterpe edulis*, with high densities of plants in the families Cyatheaceae, Rubiaceae and Rutaceae [22], [23]. Across its large geographic distribution, *E*. *edulis* has been illegally extracted for palmito, the edible apical meristem of the plant. Illegal harvesting occurred in the permanent plot after the first sampling period in 2004–2005, especially in bamboo stands, which lead to an overall decrease in *E*. *edulis* density from 202.2 to 25.7 ind/ha. As a result, marked changes in forest structure and seed rain profile occurred [24].

### Seed rain and seedling establishment

Seed rain and seedling establishment were monitored during two periods (fruiting seasons corresponding to different years, 2004–2005 and 2007–2009) in both bamboo and non-bamboo stands by using seed traps and seedling plots. From 2004 to 2005, traps and adjacent plots of 1 m^2^ each were set at 40 sampling points per stand type. From 2007 to 2009, a total of 61 sampling points received a seed trap and an adjacent seedling plot, both of 0.25 m^2^, from which 31 were in bamboo and 30 in non-bamboo stands. Sampling points were at least 20 m from each other. Seed rain was sampled monthly from Jun-2004 to Jun-2005, and from Nov-2007 to Mar-2009. Seedlings were sampled monthly from Jun-2004 to Jul-2005, and bimonthly from Jan-2008 to Nov-2008. Seeds collected in traps and seedlings identified in plots were counted and identified to the lowest taxonomic level possible (see [17] for details). Seedlings, defined as any young plant 0–30 cm height produced from a seed and consisting of a radicle, the hypocotyl, and cotyledons [25], were tagged at each census.

### Abiotic characteristics

We obtained information on variation in soil characteristics from [16] including physical (granulometry, porosity, soil density, sand, silt, and clay), and chemical parameters (pH KCl, organic matter (OM), P, K, Ca^+2^, potential acidity (H + Al), Al, and Mg). Within each sub-plot researchers in soil science collected soil samples up to 1.0 m depth with three samples per sampling point (0–5 cm, 5–20 cm and 80–100 cm). Soil samples were collected using an auger, stored in appropriate sampling containers, and sent to the Department of Soil Science at Escola Superior de Agricultura “Luiz de Queiroz” of São Paulo University, for conducting particle size and routine chemical analysis [26]. For our analysis we obtained the soil data at the specific coordinates of each sampling point with a seed trap and a seedling plot.

### Data analysis

We used species accumulation curves [27] to compare seed and seedling species richness between stands and periods (2004–2005 and 2007–2009 samplings). Datasets were prepared containing data on species abundance, for both periods at each stand type, based on the content sampled at each seed trap and seedling plot. We used the richness estimators Chao2, Jackknife 1, Jackknife 2, Bootstrap to obtain the number of expected species [28]. We used the specpool function in *vegan* package within the R environment version 3.1.2 [29], which estimates the extrapolated species richness in a species pool, or the number of unobserved species. Function specpool is based on the incidence of species in sample sites and gives a single estimate for a collection of sampling sites.

We analyzed spatial distribution patterns of seeds and seedlings by using spatial analysis by distance indices (SADIE) methodology. We chose this approach because it quantifies spatial patterns in count type data, e.g., spatially-referenced sampling units or individuals [30]. First we used SADIE index of aggregation, *I*_*a*_, to assess whether the spatial distribution of seeds and seedlings was random or clumped in bamboo and non-bamboo stands, and periods. Observed *I*_*a*_ values were compared to the distribution of *I*_*a*_ values obtained from randomized samples. We rejected the null hypothesis (randomly-generated Poisson process) if the observed value was less than 5% of the randomized values. Second, we used SADIE to test for across- and within-year concordance between the spatial distributions of seeds and seedlings. SADIE allows the identification of areas where stands of high or low density aggregate. Concordance between distributional spatial patterns can be evaluated by an association index, X [31] that ranges between +1 (complete spatial association) and -1 (complete dissociation), with 0 indicating spatial independence. This index can be tested statistically by a permutation procedure. Significance levels of X were Bonferroni corrected to account for multiple testing. SADIE analyses were conducted with the software SADIE Shell v1.22 [32].

Due to the hierarchical nature of our sampling design, i.e., the same points were sampled in two periods with a fixed number of sampling points per stand type, we used generalized linear mixed effects models (GLMM) to investigate the relationship between variation in seed and seedling richness and density in bamboo and non-bamboo stands while accounting for variation in soil variables. Response variables were seed density and richness, and seedling density and richness. Explanatory variables were stand type (bamboo or non-bamboo) and soil characteristics. For each soil variable we used the average between the first two depths (0–5 cm and 5–20 cm) at the specific coordinates of each sampling point. The depth 80–100 cm was not included in our analysis. We chose not to include this depth because it did not represent the horizon of higher absorption of nutrients and water by the seedling root system [33]. GLMMs were analyzed using the *nlme* package of R software version 3.1.2 [29].

Following the recommendations by [34], before building our GLMMs, we first checked for the presence of collinearity between soil explanatory variables and spatial autocorrelation in the response variable. We used Spearman correlation coefficient (*r* > 0.5) and variance inflation factors (VIF > 5) to assess which explanatory variables were collinear. After that, we ended up with the following selected explanatory variables: pH KCl, organic matter (OM), P, K, Ca, potential acidity (H + Al), sand, silt, and clay (see the correlation matrix of the soil characteristics in the S1 Table). These variables were then standardized to have zero mean and unit variance. To check for the presence of spatial correlation, we fitted generalized linear models (GLM), with Poisson distribution, for each response variable and all selected soil variables [35]. Then we analyzed the spatial structure of each response variable and the residuals of GLMs with spline correlograms, package *ncf* in R [35]. Spline correlograms of response variables showed clear spatial patterns at the first distance class. However, there was no spatial structure in GLM residuals in any of our models, which likely means that our explanatory variables accounted for most of the spatial structures.

Finally, we fitted the Poisson GLMMs for each response variable including all the selected soil variables and stand type as the fixed component, and period and the identity of sub-plots as the random component (when they had a significant effect in the response variable). We simplified the GLMMs by refitting them after excluding non-significant terms and checked for the presence of over-dispersion in the residuals of the models. Spline correlograms also indicated no signal of spatial structure in GLMM residuals. We used the package *lme4* [36] to fit GLMMs and the function *cftest* from the package *multcomp* [37] to assess significance of the predictors.

## Results

Seed species richness was higher in non-bamboo stands in both periods, whereas seedling species richness exhibited the same pattern in 2007–2009, but the opposite pattern in 2004–2005 (Table 1).

**Table 1:** Estimators of species richness (Chao2, Jackknife 1, Jackknife 2 and Bootstrap) for different sampling periods (2004–2005 and 2007–2009) and habitats (bamboo and non-bamboo).

	Period	Stand	Number of species	Chao2 (±SE)	Jack1 (±SE)	Jack2	Boot (±SE)
**Seed**	**2004–2005**	**Bamboo**	103	188.76 (32.61)	152.85 (17.02)	185.35	124.52 (8.15)
		**Non-bamboo**	123	195.02 (26.39)	173.77 (16.38)	204.02	145.45 (8.11)
	**2007–2009**	**Bamboo**	78	274 (87.99)	130.71 (15.11)	173.50	99.07 (6.18)
		**Non-bamboo**	122	282.10 (53.14)	195.41 (22.57)	248.52	152.38 (9.85)
**Seedling**	**2004–2005**	**Bamboo**	73	255.25 (82.43)	122.50 (29.90)	161.44	93.15 (15.59)
		**Non-bamboo**	66	164 (45.60)	104.50 (32.62)	132.68	82.01 (17.46)
	**2007–2009**	**Bamboo**	54	201 (76.20)	90(30.90)	116.43	68.89 (16.20)
		**Non-bamboo**	71	251.5 (78.02)	119.86 (46.41)	155.21	91.27 (24.82)

**Fig 1:**
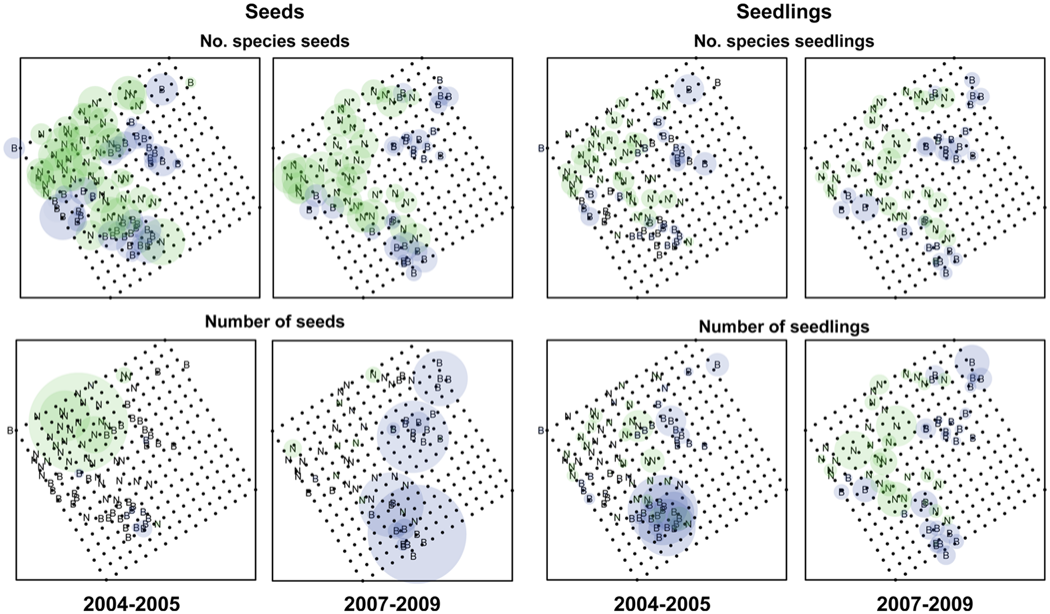
Bubble plots of seed rain and seedling density and seed and seedling richness separated by different sampling periods (2004–2005 and 2007–2009) and habitats (bamboo, B, light blue; and non-bamboo, N, light green).

Seeds and seedlings exhibited different spatial patterns in bamboo and non-bamboo stands (Fig 1). The spatial patterns of seed rain and seedling recruitment were highly aggregated, with marked variation in the intensity of aggregates and their spatial locations. The SADIE index of aggregation, *I*_*a*_, indicated that spatial patterns were very consistent across the two periods in non-bamboo stands. Within these stands, the distribution patterns of both seeds and seedlings were clearly random (Table 2). Within bamboo stands results varied, but with a tendency for spatial aggregation, as *I*_*a*_ values were high and significant (or marginally significant, i.e., *P* < 0.10; Table 2).

**Table 2:** SADIE spatial aggregation index, *I*_a_ (*P*-value), association analysis of local cluster indices of seeds and seedling in bamboo (B) and non-bamboo (NB) stands in two sampling periods.

	2004–2005		2007–2009	
	B	NB	B	NB
**Seed density**	1.43 (0.05)	0.97 (0.45)	0.89 (0.57)	0.80 (0.82)
**Seed species richness**	0.66 (0.95)	1.02 (0.41)	1.50 (0.07)	1.08 (0.32)
**Seedling density**	1.34 (0.10)	0.64 (0.99)	1.19 (0.23)	0.69 (0.93)
**Seedling species richness**	1.94 (0.01)	0.93 (0.59)	1.12 (0.29)	1.07 (0.34)

In general, the distributions of seeds and seedlings were spatially independent in both periods (2004–2005 and 2007–2009; Table 3). This result was consistent regardless of the metric (density or species richness) and stand type. We found only one case, in bamboo stands in 2004–2005, where the observed value indicated a positive spatial association (X = 0.43, P = 0.003) between the density of seeds and seedlings.

**Table 3:** SADIE spatial association analysis of local cluster indices of seeds and seedling in bamboo (B) and non-bamboo (NB) stands in two sampling periods.

	Period	Density		Species richness	
		B	NB	B	NB
**Association index**					
	2004–2005	0.43	0.26	-0.03	0.18
	2007–2009	0.34	-0.14	0.16	0.32
**Dutilleul adjusted probability level**					
	2004–2005	0.003	0.07	0.56	0.17
	2007–2009	0.06	0.73	0.21	0.07

For a Bonferroni-corrected, two-tail test with *P = 0*.*05*, the probability level should be less than 0.025 for significant association, or greater than 0.975 for significant dissociation. B stands for bamboo sites and NB for non-bamboo sites.

The final GLMMs indicated that only variation in seedling density was significantly explained by soil variables (Table 4), being positively related to OM and P, but negatively related to Ca and sand content. Seed density was positively related to pH KCL, OM and acidity (H + Al), however, we detected over-dispersion in the residuals of the GLMM describing this relationship. After adding an observation-level random effect [38], the GLMM indicated no relationship between seed density and soil variables. Variation in species richness, on the other hand, was only related to stand type, in the case of seeds (higher in non-bamboo stands). Models also indicated that only seed species richness differed between stand types while accounting for variation in soil variables; seedling species richness was, however, not related to any of the explanatory variables.

**Table 4:** Summary of final generalized linear mixed effects models for each response variable.

Seed density	Coefficient	SE	z value	*P*
-	-	-	-	-
**Seedling density**				
OM	0.16	0.06	2.80	0.005
P	0.16	0.07	2.35	0.019
Ca	-0.15	0.07	-2.11	0.035
Sand	-0.20	0.06	-3.69	< 0.001
**Seed species richness**				
Stand type	0.40	0.07	5.75	< 0.001
**Seedling species richness**				
-	-	-	-	-

## Discussion

The Atlantic forest biome is chronically and persistently disturbed by a number of factors. In addition to widespread abiotic and biotic disturbances in tropical areas (storms, tree-falls, secondary succession, overhunting of large mammals and birds), stands of bamboo can alter forest physiognomies and dynamics by adding a marked heterogeneity of microhabitat conditions for plant recruitment [20]. For animal-dispersed species these heterogeneities may represent limiting factors for regeneration, increasing seed dispersal and/or recruitment limitation [39]. In previous papers we demonstrated the influence of bamboos on the seed rain patchiness and seed limitation levels [17], as well as on the biotic factors influencing seedling establishment for several Atlantic forest species a [15]. We also showed how the harvesting of *E*. *edulis* for obtaining palm heart [40], [41], [42] that occurred after our first sampling period in 2004–2005 changed the seed rain profile at the community level [24]. Here we discuss the abiotic factors, namely soil parameters, related to seedling establishment, and focus on the spatial distribution of seeds and seedlings and how it varies between stands and sampling periods.

The clumped distribution pattern exhibited by seeds and seedlings at bamboo stands contrasts with the random pattern observed at non-bamboo stands. Such a contrast may stem from the higher seed limitation (i.e., failure of seeds to reach all potential recruitment sites) observed in bamboo stands, indicating that seed rain is spatially more heterogeneous at such stands [17]. Spatial heterogeneity in seed rain creates hotspots of seed and seedling densities, which leads to aggregation. Spatial heterogeneity in seed rain at bamboo stands may result from the lower density of trees in bamboo stands [17], [20], and the presence of emergent trees that stand out of the discontinuous and lower canopy of bamboo cover and may serve as perches for seed dispersers, thus favoring seed deposition beneath them. The presence of those relatively-isolated trees may strongly limit arrival of seeds dispersed by animal frugivores, resulting in clumped seed dissemination. An added influence on seed rain clumping is the presence of gaps in the discontinuous canopy of bamboo stands that may act as traps for the wind-dispersed seeds of pioneer plants that abounded in the study area after the harvesting of *E*. *edulis* [8], [24]. In the bamboo stand edge both seed arrival and seedling recruitment are much lower. This variation can arise from the vegetation structure combined with differences in the seed dispersal process itself.

A fundamental issue in plant demography studies is whether the initial spatial configuration of the seed rain is a good predictor of later seedling establishment (spatial concordance) [1], [10], [13]. In general seed rain pattern was discordant with seedling establishment at both bamboo and non-bamboo stands. Discordant patterns are frequently the result of post-dispersal mortality factors that limit the establishment of new individuals regardless of the number of seeds arriving in the area [12], [43]. We have shown that mortality factors are species-specific and vary between stands, causing species-specific responses to bamboo-related landscape heterogeneity [15]. In the community-wide perspective adopted here, the net effect of such variability is the overall spatial discordance between seed rain and seedling establishment. Such a discordant pattern explains why the increased seed rain that occurred in bamboo stands after the harvesting of *E*. *edulis* did not translate into greater seedling density.

### Can soil characteristics drive the distribution patterns of seeds and seedlings?

A correlation between local patterns of species distribution and soil variables has been demonstrated in several studies on tropical forests [44], [45], [46], [47]. For instance, recruitment patterns at the early seedling and sapling stages can be limited by soil nutrient availability [48]. In our study, contrary to what we expected, only seed richness differed between stand types while accounting for variation in soil characteristics. More important than soil characteristics, some studies have evidenced that post-dispersal biotic factors (e.g., seed predation and herbivory) may mediate the differences in the abundance and diversity of seeds between stands [15], [24]. [15] demonstrated that some species (*Euterpe edulis*, *Sloanea guianensis*, and *Virola bicuhyba*) are highly susceptible to predation during the early stage of the regeneration cycle. It is also expected that differences in seed species should be affected by the local vegetation structure [17], [49]. At the study area, non-bamboo stands present higher density of adult trees [17], [20], higher species density and lower concentration of small-sized trees [20]. All these features result in a complex, fine-grained, vegetation structure that creates microhabitat conditions which markedly differ from bamboo stands. Moreover, recruitment in bamboo stands shows a marked seed source limitation for certain species [17], indicating an important role for frugivore-mediated fruit removal in constraining early recruitment [48], [50].

Soil characteristics were in some cases important variables associated with spatial variation in seedling densities. Seedling densities was positively associated with organic matter content and negatively related to soil sand content and Ca concentrations. Density and richness responded to different soil conditions in the forest. This could explain most of the spatial distribution suggesting an important role of habitat filtering, accounting for seedling spatial patterns in the forest [50], where we did not reject the null hypothesis of random spatial variation.

A series of important life history processes occur in the transition from seed delivery to the adult stage. Interactions between species and environmental factors strongly influence these transitions and can significantly change the predicted model for dispersal [1], [10], [39], [51]. Thus, our results suggest that the plant community dynamics on the permanent plot is under the combined influence of stand type, vegetation structure, soil conditions, interactions between individuals and the seed rain pattern. All these factors have important consequences for plant recruitment by regulating the abundance, distribution and species diversity. Yet our results reveal that major landscape heterogeneities caused by bamboo stands can have pervasive consequences in determining plant recruitment patterns at the intermediate scale formed by the mosaic of bamboo and non-bamboo forest patches.

Knowledge of seed dispersal spatial patterns within the landscape is of great relevance for understanding the role of forest mosaics as reservoirs of plant diversity. In this context, different habitats play an important role on tropical forests regeneration and diversity maintenance. Bamboo stands are certainly important components of the landscape since both frugivore species and plant species are sensitive to environmental variables in an interactive and not separated way [52], [53]. The bamboo *G*. *tagoara* presents a large biomass allocation necessary for its functions at an initial establishment, what makes it competitive and favors its rapid colonization [54]. Also, bamboos exhibit a high plasticity in functional traits and leaf characteristics, allowing them to grow rapidly in disturbed forests [55]. Other studies revealed stands dominated by bamboos in the Atlantic forest can influence the successful recruitment of woody species [15]. Our results indicate that both stand types are influential in shaping seed rain and seedling establishment patterns in the complex forest mosaics. This suggests that hotspots of recruitment in these forests appear as dynamic as the structural changes in the vegetation related to the spread and decline of bamboo stands.

Our study highlights the importance of local microhabitat variation in shaping the community structure in tropical forests. The maintenance of diversity and spatial distribution of species is dependent on the natural forest dynamics, including bamboo dynamics. Therefore, non-natural disturbances on the forest structure caused by human activities (e.g. crops, timber or palm-heart extraction; [42]) may result in a large expansion of bamboo stands, which may lead to profound effects on local diversity by driving large-scale recruitment limitation of many plant species.

## Supporting Information

S1 Table Correlation matrix of the soil characteristics for data from Carlos Botelho State Park, São Paulo State, Brazil. (DOC)
