# Supplementary material for: Community-Wide Spatial and Temporal Discordances of Seed-Seedling Shadows in a Tropical Rainforest
Source: PLoS One. 2015 Apr 9;10(4):e0123346. doi: 10.1371/journal.pone.0123346 (PMC4391865; doi:10.1371/journal.pone.0123346)
Supplement: S1 Table — (DOC) [file pone.0123346.s001.doc]

**Supporting Information**

**Table S1.** Correlation matrix of the soil traits for data from Carlos Botelho State Park, São Paulo State, Brazil.

|  | pH_KCl | OM | P | Na | K | Ca | Al | H.Al | sandpct | siltpct |
| --- | --- | --- | --- | --- | --- | --- | --- | --- | --- | --- |
| OM | -0.291 |  |  |  |  |  |  |  |  |  |
| P | -0.520 | 0.175 |  |  |  |  |  |  |  |  |
| Na | -0.342 | 0.409 | 0.492 |  |  |  |  |  |  |  |
| K | -0.374 | 0.277 | 0.486 | 0.707 |  |  |  |  |  |  |
| Ca | 0.001 | 0.090 | 0.456 | 0.309 | 0.437 |  |  |  |  |  |
| Al | -0.573 | 0.193 | 0.271 | 0.191 | 0.033 | -0.333 |  |  |  |  |
| H.Al | -0.563 | 0.217 | 0.479 | 0.438 | 0.411 | 0.085 | 0.549 |  |  |  |
| sandpct | 0.386 | -0.075 | -0.335 | -0.420 | -0.387 | -0.123 | -0.310 | -0.370 |  |  |
| siltpct | 0.098 | -0.008 | 0.280 | 0.135 | 0.214 | 0.266 | -0.101 | 0.157 | -0.346 |  |
| natclay | -0.164 | 0.218 | 0.208 | 0.276 | 0.342 | 0.504 | -0.191 | -0.029 | -0.047 | -0.073 |
